# Editorial of *Viruses* Special Issue on Phage–Host Interactions 2021

**DOI:** 10.3390/v14020236

**Published:** 2022-01-25

**Authors:** Mikael Skurnik

**Affiliations:** 1 Department of Bacteriology and Immunology, Medicum, Human Microbiome Research Program, Faculty of Medicine, University of Helsinki, 00290 Helsinki, Finland; mikael.skurnik@helsinki.fi; 2 Division of Clinical Microbiology, HUSLAB, University of Helsinki and Helsinki University Hospital, 00290 Helsinki, Finland

*Viruses* has now published two Special Issues on phage–host interactions, the latest under the name Phage–Host Interactions 2021. In the present Special Issue we have 13 papers that study the topic from different angles. We have several papers that describe characterizations of new phages, their genomes, host ranges, identification of host receptors and receptor-binding proteins, phage proteomes and other properties. Some papers deal with conditions that influence the life cycles of phages. Additionally, prophages play different roles. 

Shchurova et al. investigated the biological properties, genomic organization, and virus–bacterial host-interaction strategy of novel myovirus TaPaz, isolated using a K47 capsule strain of *Acinetobacter baumannii.* The authors identified two different tailspike depolymerases (TSDs) and tested the purified proteins against a collection of *A. baumannii* strains belonging to 56 different K types. One TSD was identified as a specific K47 CPS glycosidase. Feng et al. described a novel Alteromonas phage vB_AmeP-R8W (R8W) that has a wide host range, mainly infecting *Alteromonas* strains isolated from deep waters. The authors show that R8W possesses two receptor-binding proteins that likely contribute to the wide host range. 

Three papers describe detailed characterizations of Yersinia phages. Happonen et al. describe the proteogenomic characterization and host-receptor identification of the siphovirus vB_YenS_φR2-01 (φR2-01) that infects strains representing several *Yersinia enterocolitica* serotypes. Of the 154 predicted genes, 117 are homologous to those of Escherichia bacteriophage T5. The outer membrane vitamin B12 receptor BtuB was identified as the host receptor of the phage. Salem et al. characterized T4-like Yersinia bacteriophages fPS-2, fPS-65, and fPS-90, isolated from pig stools, which were 84–92% identical to each other and ca 85% identical to phage T4. The phages exhibited relatively wide host ranges among *Yersinia pseudotuberculosis* and related species. Sequencing of spontaneous phage-resistant mutants revealed mutations in the *ompF*, *galU*, *hldD*, or *hldE* genes, demonstrating that the porin OmpF and the lipopolysaccharide (LPS) inner core function as phage receptors. The host recognition was assigned to the distal β-helices connecting loops of the predicted long tail-fiber tip protein, a homolog of Gp38 of T-even phages. Skurnik et al. identified *Yersinia pestis* phages fEV-1 and fD1 as a dwarf myovirus and a T4-like myovirus, respectively. They carried out proteome characterizations for both phages. fEV-1 is the first dwarf myovirus described for *Y. pestis*, and its host range was restricted strictly to *Y. pestis* strains. Phage fD1 showed wider host range, including other members of Enterobacterales such as *Escherichia coli* and *Y. pseudotuberculosis*.

Duyvejonck et al. evaluated the ability of different buffer and infusion solutions to support phage infectivity when stored at 4 °C. They observed wide variability between phages and between solutions. They also reported on the stability of lyophilized phage preparates. Hosseini et al., when working to design a phage cocktail against *Aeromonas salmonicida* subsp. *salmonicida*, the causative agent of furunculosis, noticed that when five phages were used individually, or as combinations of two to five phages, that the phage cocktail was not effective against strains bearing Prophage 3, which is present in two-thirds of the strains from the province of Quebec. Their work demonstrated that the prophage content can affect the efficacy of a cocktail of virulent phages for phage-therapy purposes. McCutcheon et al. review the clinical significance, virulence factors, and antimicrobial resistance mechanisms of *Stenotrophomonas maltophilia*, all the phages isolated and characterized to date, and strategies for their use. They describe the in vivo phage-therapy studies conducted against this bacterium and discuss future prospects for the use of phages to treat multidrug-resistant *S. maltophilia*. Zhang et al. isolated a T4-like lytic phage targeting *Escherichia coli* C600 carrying plasmid pHNAH67 encoding 11 antibiotic resistance genes (ARGs). Using NGS they found that the phage could misload host DNA randomly, including the plasmid ARGs.

Hünnefeld et al. characterized the genome of Corynebacterium phage CL31 and analyzed its infection dynamics in the *Corynebacterium glutamicum* strain ATCC 13032. CL31 efficiently infected a mutant strain lacking the restriction-modification system, and even more efficiently the prophage-free *C. glutamicum* strain MB001. The authors furthermore carried out proteome analysis of purified phage particles, and transcriptome analysis of infected bacteria. Analysis of CL31-resistant strains revealed SNPs in genes involved in mycolic acid biosynthesis, suggesting a role of this cell envelope component in phage adsorption. Bastin et al. report that phage MS2 when propagated in *Escherichia coli* under aerobic conditions produces mostly non-infectious phage particles. Indeed, only 2% of the produced phage particles were infectious. They show that growth under anaerobic conditions and ROS inhibition increases infectious phages to 8 and 25%, respectively. Thus, the authors predict that oxidative damage impacts the molecular replication and assembly of MS2 phages. Sacher et al. describe the characterization of a *Campylobacter jejuni* phage that requires functional flagellar glycosylation and motor genes for infection, although the flagellae are not necessary for adsorption of the phage on the cell surface. The authors identify, using sophisticated approaches, a link between oxidative stress, flagellar motility, and phage infectivity, demonstrating that the host oxidative stress state is an important issue to be taken into account in phage–host interaction studies. Skliros et al., using a multiomics approach incorporating transcriptome and metabolome analyses of *Vibrio alginolyticus* phage-resistant strains, showed that the strains had repressed the expression of the *ompF*, *lamB*, and *btuB* genes encoding phage receptors. However, this was accompanied by repression of the genes encoding natural nutrient channels, such as RbsA, PtsG, TryP, LivH, LysE, and HisP. This suggests that the bacterial cell needs to readjust its biochemistry to achieve phage resistance. Lavysh et al. report that Salmonella phage 7–11, similarly to Escherichia phage phiEco32, encodes a protein similar to the bacterial sigma subunit of RNA polymerase. They investigated the temporal pattern of phage 7–11 gene expression during infection and compared it to the previously determined transcription strategy of phiEco32. Using sophisticated methods, they identified eight promoters recognized by host RNA polymerase holoenzyme equipped with the phage 7–11 sigma subunit. These promoters are characterized by a bipartite consensus GTAAtg-(16)-aCTA and are located upstream of late phage genes. Interestingly, the transcription profiles of the two related phages differ from each other, likely due to the fact that phage 7–11 does not encode the inhibitor of host transcription and early phage transcription or the activator of transcription.

